# Supplementary material for: Persistent activity in a recurrent circuit underlies courtship memory in Drosophila
Source: eLife. 2018 Jan 11;7:e31425. doi: 10.7554/eLife.31425 (PMC5800849; doi:10.7554/eLife.31425)
Supplement: Supplementary file 1. — Constitutive silencing of MBγ neurons impairs STM. Courtship indices of naïve (CI-) and experienced (CI+) males of the indicated genotypes according to Figure 1A, tested in single-pair assays with mated females as trainers and testers, shown as mean ± s.e.m. and median (italics) of n males. P values determined by permutation test for the null hypothesis that learning equals 0 (H0: SI = 0) or for the null hypothesis that experimental and control males learn equally well (H0: SI = SIc). Table S2. Constitutive silencing of M6 neurons impairs STM. Courtship indices of naïve (CI-) and experienced (CI+) males of the indicated genotypes according to Figure 1B, tested in single-pair assays with mated females as trainers and testers, shown as mean ± s.e.m. and median (italics) of n males. P values determined by permutation test for the null hypothesis that learning equals 0 (H0: SI = 0) or for the null hypothesis that experimental and control males learn equally well (H0: SI = SIc). Table S3. Activation of MBγ neurons is more potent in experienced than naïve males. Courtship indices at 20⁰C (CI20) and 32⁰C (CI32) of naïve (-) or experienced (+) males of the indicated genotypes according to Figure 1C, tested in single-pair assays with pseudomated females, shown as mean ± s.e.m. and median (italics) of n males. All males where trained at room temperature. P values determined by permutation test for the null hypotheses that learning equals 0 (H0: SI = 0), that experimental flies do not differ from the controls (H0: SI = SIc), and that courtship is equally suppressed in experienced and naïve males (H0: SI+=SI-). Table S4. Activation of M6 neurons is equally potent in naïve and experienced males. Courtship indices at 20⁰C (CI20) and 32⁰C (CI32) of naïve (-) or experienced (+) males of the indicated genotypes according to Figure 1D, tested in single-pair assays with pseudomated females, shown as mean ± s.e.m. and median (italics) of n males. All males where trained at room tempera [file elife-31425-supp1.docx]

**Supplementary File 1. Tables S1-S4**

**Table S1. Constitutive silencing of MBγ neurons impairs STM**

| **Genotype**  **GAL4 / UAS-TNT** | **CI^-^ (%)** | **n** | **CI^+^ (%)** | **n** | **SI (%)** | ***P SI=0*** | ***P SI=SI_c_*** |
| --- | --- | --- | --- | --- | --- | --- | --- |
| VT044966/TNT | 35.6±1.9  *34.7* | 56 | 36.4±2.0  *34.2* | 62 | -2.3  *1.5* | 0.62  *0.43* | 0.0002  *0.0012* |
| VT044966/TNTQ | 35.6±1.9  *34.7* | 56 | 20.4±1.7  *15.3* | 59 | 42.6  *55.8* | $<$0.0001  $<$*0.0001* |  |
| VT030413/TNT | 31.1±1.2  *30.3* | 67 | 34.5±2.1  *32.5* | 70 | -10.8  *-6.2* | 0.92  *0.70* | $<$0.0001  $<$*0.0001* |
| VT030413/TNTQ | 37.6±2.0  *36.8* | 64 | 21.5±1.8  *18.4* | 63 | 42.9  *48.5* | $<$0.0001  $<$*0.0001* |  |

Courtship indices of naïve (CI^-^) and experienced (CI^+^) males of the indicated genotypes according to Figure 1A, tested in single-pair assays with mated females as trainers and testers, shown as mean ± s.e.m. and median (italics) of *n* males. *P* values determined by permutation test for the null hypothesis that learning equals 0 (H_0_: SI = 0) or for the null hypothesis that experimental and control males learn equally well (H_0_: SI = SI_c_).

**Table S2. Constitutive silencing of M6 neurons impairs STM**

| **Genotype**  **GAL4 / UAS-TNT** | **CI^-^ (%)** | **n** | | **CI^+^ (%)** | **n** | **SI (%)** | ***P SI=0*** | ***P SI=SI_c_*** |
| --- | --- | --- | --- | --- | --- | --- | --- | --- |
| VT014702/TNT | 46.2±2.1  *46.3* | 50 | | 28.9±1.9  *25.3* | 53 | 37.5  *45.4* | $<$0.0001  $<$*0.0001* | 0.0021  *0.033* |
| VT014702/TNTQ | 47.8±1.9  *46* | 54 | | 20.5±1.4  *17.5* | 52 | 57.1  *62.0* | $<$0.0001  $<$*0.0001* |  |
| VT032411/TNT | 26.7±2.2  *25.4* | 54 | 22.4±1.7  *19.0* | | 54 | 17.1  *25.1* | $<$0.0001  $<$*0.0001* | $<$0.0005  $<$*0.0042* |
| VT032411/TNTQ | 36.0±1.6  *34.1* | 54 | | 17.5±1.7  *15.3* | 51 | 51.2  *55.2* | $<$0.0001  $<$*0.0001* |  |

Courtship indices of naïve (CI^-^) and experienced (CI^+^) males of the indicated genotypes according to Figure 1B, tested in single-pair assays with mated females as trainers and testers, shown as mean ± s.e.m. and median (italics) of *n* males. *P* values determined by permutation test for the null hypothesis that learning equals 0 (H_0_: SI = 0) or for the null hypothesis that experimental and control males learn equally well (H_0_: SI = SI_c_).

**Table S3. Activation of MBγ neurons is more potent in experienced than naïve males**

| **Genotype** | **train** | **CI^20^ (%)** | **n** | **CI^32^ (%)** | **n** | **SI (%)** | ***P SI=0*** | ***P SI=SI_c_*** | ***P SI^+^=SI^-^*** |
| --- | --- | --- | --- | --- | --- | --- | --- | --- | --- |
| VT044966-GAL4/UAS-TrpA1 | - | 51.4±3.27  *49.5* | 49 | 47.6±5.36  *44.9* | 37 | 7.4  *9.3* | 0.26  *0.22* |  |  |
| VT044966-GAL4/+ | - | 51.1±2.22  *52.3* | 49 | 57.5±4.25  *63.7* | 45 | -12.6  *-21.8* | 0.91  *0.99* | 0.19  *0.21* |  |
| UAS-TrpA1/+ | - | 49.3±3.11  *50.4* | 47 | 57.6±5.5  *63.1* | 31 | -16.8  *-25.1* | 0.92  *0.98* | 0.18  *0.22* |  |
| VT044966-GAL4/UAS-TrpA1 | + | 44.5±4.0  *48.6* | 39 | 23.6±4.42  *9.3* | 39 | 47.0  *80.9* | 0.0005  $<$*0.0001* |  | 0.005  *0.001* |
| VT044966-GAL4/+ | + | 40.5±4.22  *38.1* | 37 | 46.3±5.28  *48.6* | 33 | -14.4  *-27.7* | 0.80  *0.78* | $0.002$  *0.002* | 0.16  *0.27* |
| UAS-TrpA1/+ | + | 43.9±4.8  *44.3* | 30 | 43.0±5.4  *43.7* | 29 | 2.1  *9.0* | 0.46  *0.40* | 0.002  *0.002* | 0.70  *0.72* |

Courtship indices at 20⁰C (CI^20^) and 32⁰C (CI^32^) of naïve (-) or experienced (+) males of the indicated genotypes according to Figure 1C, tested in single-pair assays with pseudomated females, shown as mean ± s.e.m. and median (italics) of *n* males. All males where trained at room temperature. *P* values determined by permutation test for the null hypotheses that learning equals 0 (H_0_: SI = 0), that experimental flies do not differ from the controls (H_0_: SI = SI_c_), and that courtship is equally suppressed in experienced and naïve males (H_0_: SI^+^ = SI^-^).

**Table S4. Activation of M6 neurons is equally potent in naïve and experienced males**

| **Genotype** | **train** | **CI^20^ (%)** | **n** | **CI^32^ (%)** | **n** | **SI (%)** | ***P SI=0*** | ***P SI=SI_c_*** | ***P SI^+^=SI^-^*** |
| --- | --- | --- | --- | --- | --- | --- | --- | --- | --- |
| VT032411-GAL4/UAS-TrpA1 | - | 45.6±2.3  *47.1* | 54 | 24.6±3.3  *15.4* | 53 | 47.0  *73.4* | $<$0.0001  $<$*0.0001* |  | 0.20  *0.34* |
| VT032411-GAL4/+ | - | 50.5±3.0  *55.8* | 44 | 56.4±3.5  *59.3* | 44 | -9.3  *-5.4* | 0.81  *0.25* | $<$0.0001  *0.0002* | 0.47  *0.76* |
| UAS-TrpA1/+ | - | 39.3±4.2  *43.5* | 37 | 44.8±4.4  *36.8* | 37 | -14.0  *15.3* | 0.78  *0.26* | 0.0002  *0.002* | 0.06  *0.45* |
| VT032411-GAL4/UAS-TrpA1 | + | 43.4±2.2  *40.9* | 59 | 17.6±2.4  *8.5* | 59 | 59.4  *79.3* | $<$0.0001  $<$*0.0001* |  |  |
| VT032411-GAL4/+ | + | 46.1±2.7  *45.4* | 44 | 42.6±3.8  *46.0* | 44 | 7.5  *-3.1* | 0.23  *0.65* | $<$0.0001  $<$*0.0001* |  |
| UAS-TrpA1/+ | + | 39.4±2.9  *42.7* | 40 | 29.6±3.4  *27.5* | 48 | 24.4  *35.5* | 0.02  *0.01* | 0.001  *0.012* |  |

Courtship indices at 20⁰C (CI^20^) and 32⁰C (CI^32^) of naïve (-) or experienced (+) males of the indicated genotypes according to Figure 1D, tested in single-pair assays with pseudomated females, shown as mean ± s.e.m. and median (italics) of *n* males. All males where trained at room temperature. *P* values determined by permutation test for the null hypotheses that learning equals 0 (H_0_: SI = 0), that experimental flies do not differ from the controls (H_0_: SI = SI_c_), and that courtship is equally suppressed in experienced and naïve males (H_0_: SI^+^ = SI^-^).
