# Supplementary material for: Persistent activity in a recurrent circuit underlies courtship memory in Drosophila
Source: eLife. 2018 Jan 11;7:e31425. doi: 10.7554/eLife.31425 (PMC5800849; doi:10.7554/eLife.31425)
Supplement: Supplementary file 2. — Acute silencing of M6 neurons impairs STM acquisition and retrieval. Courtship indices of naïve (CI-) and experienced (CI+) males of the indicated genotypes according to Figure 4H, tested in single-pair assays at the indicated temperature (⁰C) during training (Train) or testing (Test), with mated females as trainers and testers, shown as mean ± s.e.m. and median (italics) of n males. P values determined by permutation test for the null hypothesis that learning equals 0 (H0: SI = 0) or for the null hypothesis that experimental and either type of control males learn equally well (H0: SI = SIc). [file elife-31425-supp2.docx]

**Supplementary File 2. Table S5**

**Table S5 Acute silencing of M6 neurons impairs STM acquisition and retrieval**

| **Genotype** | **Train** | **Test** | **CI^-^ (%)** | **n** | **CI^+^ (%)** | **n** | **SI (%)** | ***P SI=0*** | ***P SI=SI_c_*** |
| --- | --- | --- | --- | --- | --- | --- | --- | --- | --- |
| VT032411-GAL4/UAS-shi^ts^ | 32⁰C | 22⁰C | 55.9±2.5  *56.5* | 52 | 52.9±2.4  *58.1* | 51 | 6.2  *-1.5* | $0.20$  $0.54$ |  |
| VT032411-GAL4/+ | 32⁰C | 22⁰C | 41.9±2.0  *40.0* | 48 | 26.3±3.3  *23.0* | 45 | 37.3  *42.5* | 0.01  *0.002* | 0.005  *0.013* |
| UAS-shi^ts^/+ | 32⁰C | 22⁰C | 29.2±3.3  *28.2* | 35 | 15.4±2.6  *6.0* | 47 | 47.3  *78.6* | 0.005  *0.04* | 0.0039  *0.0003* |
| VT032411-GAL4/UAS-shi^ts^ | 22⁰C | 32⁰C | 44.4±2.8  *46.6* | 34 | 39.0±2.6  *40.9* | 49 | 12.2  *13.0* | 0.08  *0.04* |  |
| VT032411-GAL4/+ | 22⁰C | 32⁰C | 40.5±2.9  *43.05* | 30 | 25.0±2.7  *18.3* | 46 | 38.2  *58.1* | $<$0.0001  $<$*0.0001* | 0.009  *0.056* |
| UAS-shi^ts^/+ | 22⁰C | 32⁰C | 30.7±2.6  *31.27* | 30 | 19.6±2.1  *15.0* | 43 | 36.1  *51.6* | 0.004  *0.003* | 0.0013  *0.0004* |

Courtship indices of naïve (CI^-^) and experienced (CI^+^) males of the indicated genotypes according to Figure 4H, tested in single-pair assays at the indicated temperature (⁰C) during training (Train) or testing (Test), with mated females as trainers and testers, shown as mean ± s.e.m. and median (italics) of *n* males. *P* values determined by permutation test for the null hypothesis that learning equals 0 (H_0_: SI = 0) or for the null hypothesis that experimental and either type of control males learn equally well (H_0_: SI = SI_c_).
