# Supplementary material for: Persistent activity in a recurrent circuit underlies courtship memory in Drosophila
Source: eLife. 2018 Jan 11;7:e31425. doi: 10.7554/eLife.31425 (PMC5800849; doi:10.7554/eLife.31425)
Supplement: Supplementary file 3. — Specific fly genotypes used in all main and supplementary figures. [file elife-31425-supp3.docx]

**Supplementary File 3. Fly genotypes**

**Figure 1A**

*+/Y; UAS-TNT/+; VT044966-GAL4/+*

*+/Y; UAS-TNTQ/+; VT044966-GAL4/+*

*+/Y; UAS-TNT/+; VT030413-GAL4/+*

*+/Y; UAS-TNTQ/+; VT030413-GAL4/+*

Trainer and tester females: Canton-S, mated to Canton-S males

**Figure 1B**

*+/Y; UAS-TNT/+; VT014702-GAL4/+*

*+/Y; UAS-TNTQ/+; VT014702-GAL4/+*

*+/Y; UAS-TNT/+; VT032411-GAL4/+*

*+/Y; UAS-TNTQ/+; VT032411-GAL4/+*

Trainer and temales: Canton-S, mated to Canton-S males

**Figure 1C**

*+/Y; UAS-TrpA1/+; VT044966-GAL4/+*

*+/Y; + /+; VT044966-GAL4/+*

*+/Y; UAS-TrpA1/+; +/+*

Tester females: *+; elav-GAL4/+; UAS-SP/+* (pseudomated) virgins

**Figure 1D**

*+/Y; UAS-TrpA1/+; VT032411-GAL4/+*

*+/Y; + /+; VT032411-GAL4/+*

*+/Y; UAS-TrpA1/+; +/+*

Tester females: *+; elav-GAL4/+; UAS-SP/+* (pseudomated) virgins

**Figure 1 - Figure supplement 1**

*w1118/Y; UAS-mCD8-GFP/+; VT044966-GAL4/+*

*w1118/Y; UAS-mCD8-GFP/+; VT030413-GAL4/+*

*w1118/Y; UAS-mCD8-GFP/+; VT014702-GAL4/+*

*w1118/Y; UAS-mCD8-GFP/+; VT032411-GAL4/+*

*w1118/Y; VT014702-LexAGAD/+; LexAop-mCD8-GFP/+*

*w1118/Y; UAS-mCD8-GFP/+; VT045584-GAL4/+*

*w1118/Y; VT006202-LexAGAD/+; LexAop-mCD8-GFP/+*

*w1118/Y; UAS-mCD8-GFP/+; VT06202-GAL4/+*

**Figure 2**

*+/Y; VT014702-LexAGAD/UAS-SFOCatCh; VT044966-GAL4/LexAop2-GCaMP6s-p10*

**Figure 2 - Figure supplement 1**

*w1118/Y; GH146-GAL4/UAS-SFOCatCh; UAS-mCD8-GFP/+*

**Figure 2 - Figure supplement 2**

*w1118/Y; VT014702-LexAGAD/UAS-SFOCatCh; VT044966-GAL4/LexAop2-GCaMP6s-p10*

**Figure 2 - Figure supplement 3**

*w1118/Y; VT014702-LexAGAD/LexAop2-opGCaMP6s; VT044966-GAL4/UAS-CsChrimson-tdTomato*

**Figure 3A-D**

*+/Y; VT014702-LexAGAD/UAS-SFOCatCh; VT044966-GAL4/LexAop2-GCaMP6s-p10*

*+/Y; VT014702-LexAGAD/+; VT044966-GAL4/LexAop2-GCaMP6s-p10*

**Figure 3E-F**

*w^1118^/Y; VT006202-LexAGAD/UAS-SFOCatCh; VT044966-GAL4/LexAop2-GCaMP6s-p10*

**Figure 4A-D**

*+/Y; VT06202-LexAGAD/UAS-SFOCatCh; VT045584-GAL4/LexAop2-GCaMP6s-p10*

**Figure 4E-G**

*+/Y; VT06202-LexAGAD/UAS-SFOCatCh; VT044966-GAL4/LexAop2-GCaMP6s-p10*

**Figure 4I**

*+/Y; UAS-shi*^ts^*/+; VT032411-GAL4/+*

*+/Y; +/+; VT032411-GAL4/+*

*+/Y; UAS-shi*^ts^*/+; +/+*

Trainer and tester females: Canton-S, mated to Canton-S males

**Figure 4 - Figure supplement 1**

*w*^1118^*/Y; UAS-SFOCatCh; VT006202-GAL4/UAS-GCaMP6s-p10*

**Figure 4 - Figure supplement 2**

*w*^1118^*/Y; VT014702-LexAGAD/UAS-SFOCatCh; VT044966-GAL4/LexAop2-GCaMP6s-p10*
